# Osteopathology and selenium deficiency co-occurring in a population of endangered Patagonian huemul (*Hippocamelus bisulcus*)

**DOI:** 10.1186/s13104-015-1291-9

**Published:** 2015-08-02

**Authors:** Werner T Flueck

**Affiliations:** National Council of Scientific and Technological Research (CONICET), Buenos Aires, Argentina; Argentine National Park Administration, Bariloche, Argentina; Swiss Tropical and Public Health Institute, University Basel, Basel, Switzerland; Chair, Scientific Committee, IUCN Huemul Task Force, C.C. 592, 8400 Bariloche, Argentina

**Keywords:** Huemul, *Hippocamelus bisulcus*, Selenium deficiency, Osteopathology, Lack of recovery

## Abstract

**Background:**

About 1,000 endangered Patagonian huemul deer (*Hippocamelus bisulcus*) remain in Chile and 350–500 in Argentina. Most groups (>100) are not recovering, and prevalence of osteopathology in Argentina was at least 57%. Here I describe relevant cases of osteopathology from a Chilean population which, however, recently also provided data on trace mineral status, supporting the initial hypothesis that nutrition may be a primary etiologic factor. Additionally, recent data on bone chemical composition of Argentine cases and soil analyses are discussed.

**Results:**

Fluoride levels in Argentine cases with osteopathology were low and fluorosis was discarded as an etiological factor. Selenium deficiency occurred in 73% of huemul from the Chilean population which exhibited several cases with osteopathology. The pathophysiognomy included extensive erosion; tooth loss; porosification; perforations of palate, maxillar and mandibular bone with frequent exposure of tooth roots; and fractured mandibula. Areas currently used by remaining huemul have mainly acidic volcanic soils, which reduces selenium bioavailability: mean soil selenium levels from areas typically used by extant huemul were very deficient (0.19 ppm), corroborating documented overt selenium deficiency in local livestock and plants. The area of extant huemul is known to result in primary iodine deficiency in livestock which is aggravated by selenium deficiency.

**Conclusions:**

Currently the most parsimonious explanation for frequent osteopathology and lack of numerical recovery are the combined effects of selenium and iodine deficiencies based on: osteopathology in a population of selenium deficient huemul; selenium deficient livestock, plants and soils; acidic soils; and regional primary iodine deficiency. The nexus between mineral nutrition and population dynamics of huemul may be due to constraints on their movements to fertile lowlands, including the elimination of historic migratory traditions, and concomitant elimination of source populations.

## Background

The Patagonian huemul (*Hippocamelus bisulcus*), a cervid endemic to the Southern Cone of Latin America, has been considered endangered for over a century [1], yet their numbers continue to decline and there may be as few as 1,500 individuals remaining. Habitat conditions for the species include wet temperate rain forests to the west, and, due to the Andean mountains’ rain shadow, drier forests and grasslands to the east. The latter habitat types occur mainly in Argentina. Huemul numbers and area of occupancy likely began to decline after the increase in numbers of pre-Columbian tribes, and accounts by the earliest explorers about huemul from interior Patagonia thus described a landscape that had been modified already for several hundred years [1–3]. Based on the evidence of historical and archaeological data, huemul have also occupied open vegetation zones such as the steppe far from forests [4–6]. Scientific studies on Argentine huemul started in the mid-1980 s, revealing that only 350–500 animals are remaining. The knowledge base on huemul was rudimentary, and a CrossSearch of ISI Web-of-Knowledge (http://isiknowledge.com) and 17 external databases (1945–2006) listed 16 entries on *H. bisulcus*, with only nine original studies [7]. Given the lack of knowledge on the role of disease in reduced huemul populations, a study focusing on skeletal remains of Argentine huemul provided essential baseline data on bone diseases and evaluated the potential of disease to contribute to the species’ morbidity [2]. The prevalence of osteopathy among adults was at least 57%, because a third of the cases were inconclusive due to <3 available bones. Among affected individuals, 63% showed mandibular, 100% maxillary, and 78% appendicular lesions. Although predation resulted in most of those death, the observed skeletal lesions would affect predator avoidance, possibly explaining the low average adult age (3.1 year) and lack of population recovery. Several primary etiologic factors were discarded: senescence; gender; fulminating infections; congenital anomalies; metabolic, endocrine, genetic, or neurologic disorders; parasitism or marasmus; and fluorosis. Instead, generalized secondary chronic alveolar osteomyelitis and osteoarthritis in huemul was hypothesized to relate to the nutritional ecology of these animals: selenium (Se) deficiency, which impairs bone metabolism and causes periodontitis in ruminants, occurs in the region and it is more prevalent at high altitudes where extant huemul tend to be confined. The new information presented here lends strong support for the hypothesized causal relationship between nutritional constraints and population health and dynamics.

The paper aims at amending the discussion about the prevalent occurrence of osteopathology in huemul with relevant new data, while recognizing that the predicament of this species hinges on an as of yet limited base of scientific understanding of factors which have prevented the recovery of most of the >100 fragmented subpopulations in Chile and Argentina.

## Potential etiologic factors causing osteopathology in huemul

### Fluorosis

Fluorosis results also in strikingly similar patterns of secondary skull infections and osteolytic processes, and chronic fluoride intoxication causes hyperostosis in the postcranial skeleton and hoof deformities [8]. The Andes are volcanically very active, and fluorides have been shown to be deposited at hundred of kilometers from volcanic sources causing fluorosis [9, 10]. However, as there were no known fluorosis cases among people nor livestock near the study sites of the affected huemul reported in 2008, fluorosis as a primary cause was discarded [2]. However, based on recent analyses of bone fluoride levels in these huemul afflicted with osteopathology [2], fluorosis was now confirmed not to have been a factor, as the average fluorine level was only 58 ppm (SE = 10.7) [10]. In comparison, exposure to fluorine-containing volcanic ashes has resulted in mild dental fluorosis in local deer with bone fluoride levels of 700–800 ppm, and severe dental fluorosis with 1,500–2,500 ppm F [10, 11], whereas >4,000 ppm F has resulted in osteofluorotic alterations in deer [12].

### Selenium deficiency

Selenium deficiency not only reduces host defense mechanisms but also impairs bone metabolism, causing osteopenia and osteoarthritis [13, 14]. In similar environments of New Zealand, Se deficiency in ruminants was shown to be the underlying factor for periodontitis, mandibular thickening, premature tooth shedding, and reduced bone density [15, 16], similarly to the lesions described in the present paper and in huemul from Argentina [2].

Southern Chile, which coincides with the huemul distribution, is known to result in Se deficient livestock [17–19]. Also, Se concentrations in most forages in southern Chile are deficient [20] and this deficiency is associated with overt pathology in livestock including white muscle disease [21]. Similarly, the first-ever study of the Se status in huemul was done in the subpopulation of the Chilean Reserva Nacional Lago Cochrane (RNLC, 47.214806°S, 72.509129°W) [22], revealing that 73% of huemul were deficient (64% severely deficient) [23] as compared to reference values as determined for deer and cattle [24, 25].

**Fig. 1:**
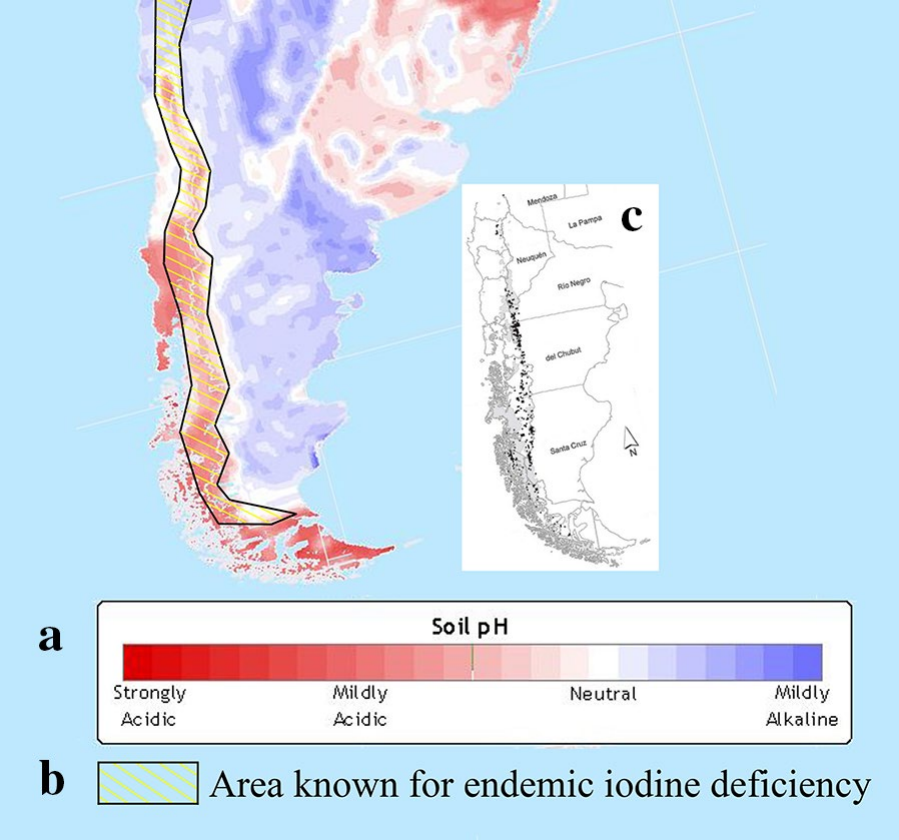
Patterns of soil acidity and iodine deficiency in relation to the extant huemul distribution. **a** Soil acidity in southern South America, from the Atlas of the Biosphere, Univ. Wisconisn, www.sage.wisc.edu/atlas/index.php: data taken from IGBP-DIS Global Soils Dataset, 1998. **b** Distribution of primary iodine deficiency [28, 29]. **c** Current distribution of huemul subpopulations: each *square* represents a huemul group mostly within 8 × 8 km or less.

Bioavailability of Se is reduced in more acidic soils by transforming Se into unusable forms unavailable for plant uptake [26, 27]. In addition, the export of plant biomass by harvesting and removing it from the site, either directly or indirectly via herbivores, removes important proportions of bioavailable Se and also results in further soil acidification [25]. Significantly, extant huemul currently inhabit principally areas that contain volcanic soils which in fact are mainly acidic (Fig. 1).

Areas elsewhere providing very low or low Se in forage are characterized by volcanic and basaltic rocks, granite, argillites and quartzites, andesitic and puroclastic rocks, glacial deposits, and cold-humid soils, and thus, most extant huemul groups likely exist in areas of Se deficient soils [30–32]. Moreover, volcanic ashes as main contributor to soil formation would contain low levels of Se compounds due to having volatilized during volcanic activity [33, 34]. Lastly, recent analyses of soils from several areas as used by extant huemul clearly showed deficient Se level, averaging only 0.19 ppm (SE = 0.02, n = 12) [35], when concentrations of 0.1–0.6 ppm Se are considered deficient [31, 32].

### Iodine deficiency

Iodine deficiency in southern Chile has been documented in people [28] and livestock [36, 37]. Similarly, in areas with huemul along the eastern side of the Andes, the goiter rates in 1965 among 20 year-old men in two provinces were 33 and 48% [38]. Therefore, the areas recognized for endemic iodine deficiency coincides with the distribution of the remaining extant huemul groups (Fig. 1).

Primary iodine deficiency thus may also play a role for huemul. Moreover, it is aggravated additionally by Se deficiency causing secondary iodine deficiency, as Se has an essential role in thyroid hormone metabolism, and thus plays a major role in the outcome of iodine deficiency. First, there are three Se-containing deiodinases which regulate the synthesis and degradation of triiodothyronine (T3), a biologically active thyroid hormone required for normal growth and development, and for energy production and O_2_ consumption in cells. Second, selenoperoxidases and possibly thioredoxin reductase protect the thyroid gland from H_2_O_2_ produced during the synthesis of thyroid hormones [39, 40]. For instance, Se supplementation in Se deficient cattle resulted in higher T3 activity and higher IG concentrations in colostrum [41, 42].

## Osteopathology and selenium deficiency in the same huemul population

The dispute over the recent analysis of the Se blood data from huemul in the RNLC [22, 23] prompted us to review all the information gathered during our research conducted in the RNLC on that same huemul population in 1993. At that time it was our first exposure to live huemul which thus were the focus of the study. Yet remains of several dead huemul were also found, revised, and documented photographically, but these results have not been published before. The review of our notes and diapositive slides revealed the existence of several cases of huemul affected with pronounced osteopathology. This material revised during our 1993 study does not allow to estimate the disease prevalence, but mainly serves to indicate the repeated occurrence of this disease pattern in the RNLC, and to document the degree of disease severity in that population.

### Description of the cases in the Reserva Nacional Lago Cochrane afflicted with osteopathology

The following cases were found during our 1993 study. There may be more cases in materials collected in RNLC by colleagues before and after 1993, but to my knowledge there have been no previous reports of osteopathology in huemul from RNLC.

**Fig. 2:**
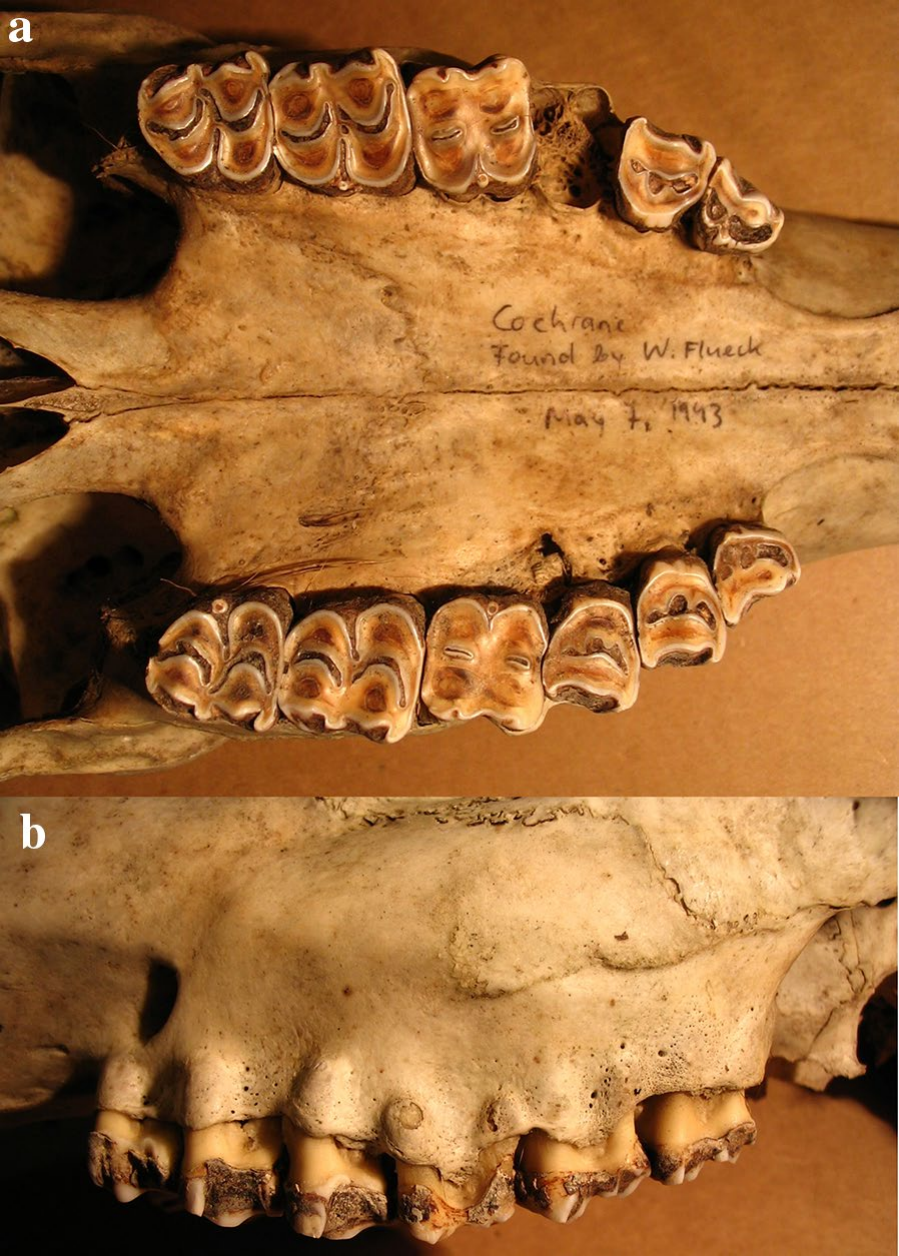
Case 1, a female 6–8 years old. **a** Extensive erosion of alveoli and thus missing P4, erosions of the palate with porosification and perforations. **b** Porosification and perforations on left maxillary buccal side exposing root apices.

Case 1 is a skull from a female estimated at 6–8 years old based on tooth wear. The right maxillary P4 is missing, likely due to the extensive erosion of the alveoli (Fig. 2a). At the level of the left P4 and M1, the palate shows erosions resulting in porosification and even perforations (Fig. 2a). The left buccal side of the maxilla show porosification at P3, M2 and M3, whereas perforations at M1 expose the roots (Fig. 2b).

**Fig. 3:**
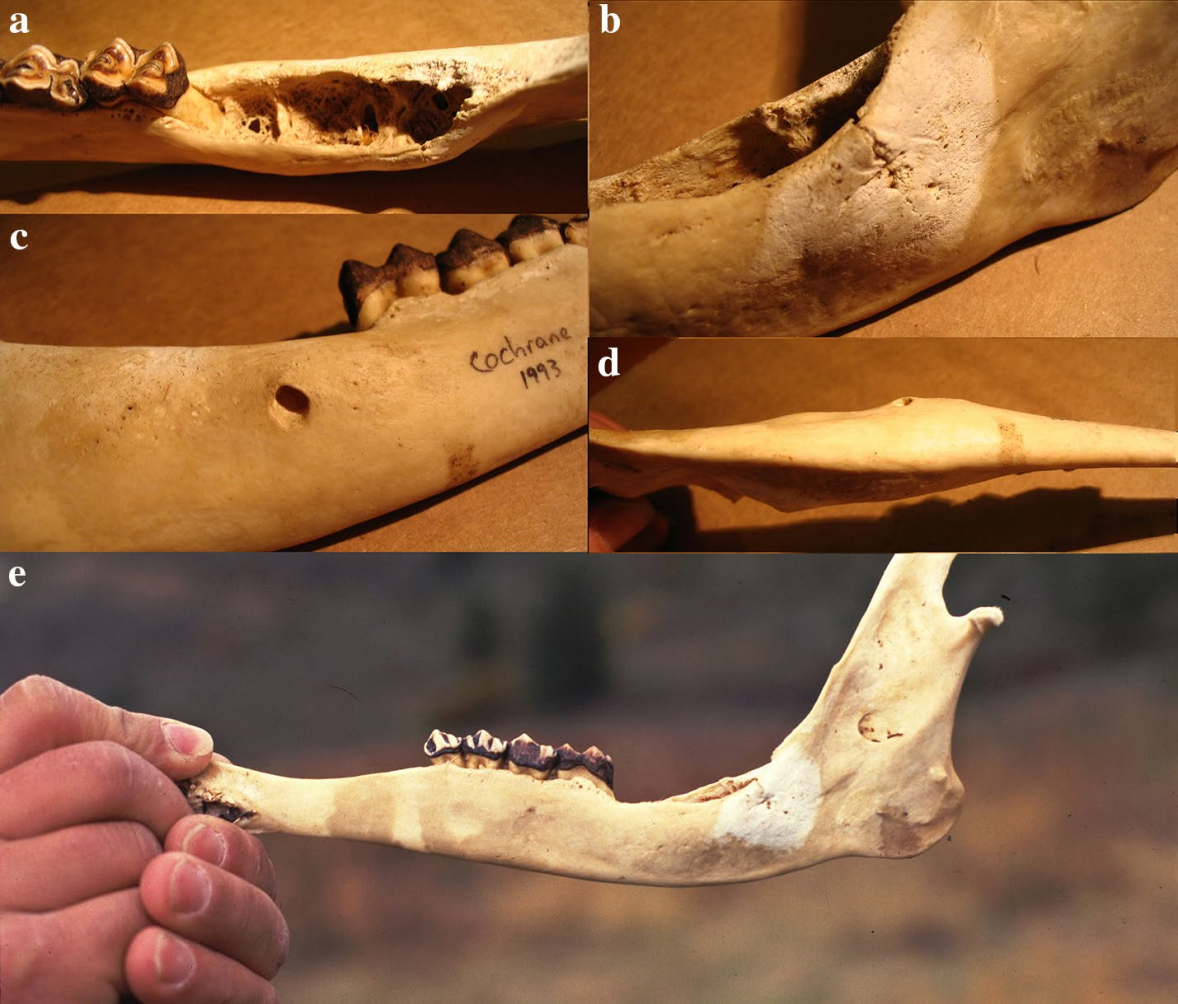
Case 2, a huemul 6–8 years old. **a** Extensive erosion of dental alveoli with M2 and M3 missing. **b** Pronounced porosification by M3 causing a pathological fracture of approximately half the mandibular height. **c** Osteolytic lesion causing a large perforation. **d** Bilateral thickening of mandibular body. **e** Bone resorption at upper margin reducing mandibular height.

Case 2 is the right half of a mandible (huemul 6–8 years old), with extensive erosion of alveolar pockets of M2 and M3, and loss of both teeth (Fig. 3a). The process of porosification at the level on M3 is so pronounced that it caused a fracture on the buccal side of approximately half the mandibular height (Fig. 3b). On the buccal side of M3, the process caused an osteolytic lesion in form of a large perforation (Fig. 3c), and while this portion of the mandible underwent substantial thickening (Fig. 3d), its height was reduced substantially (Fig. 3e).

**Fig. 4:**
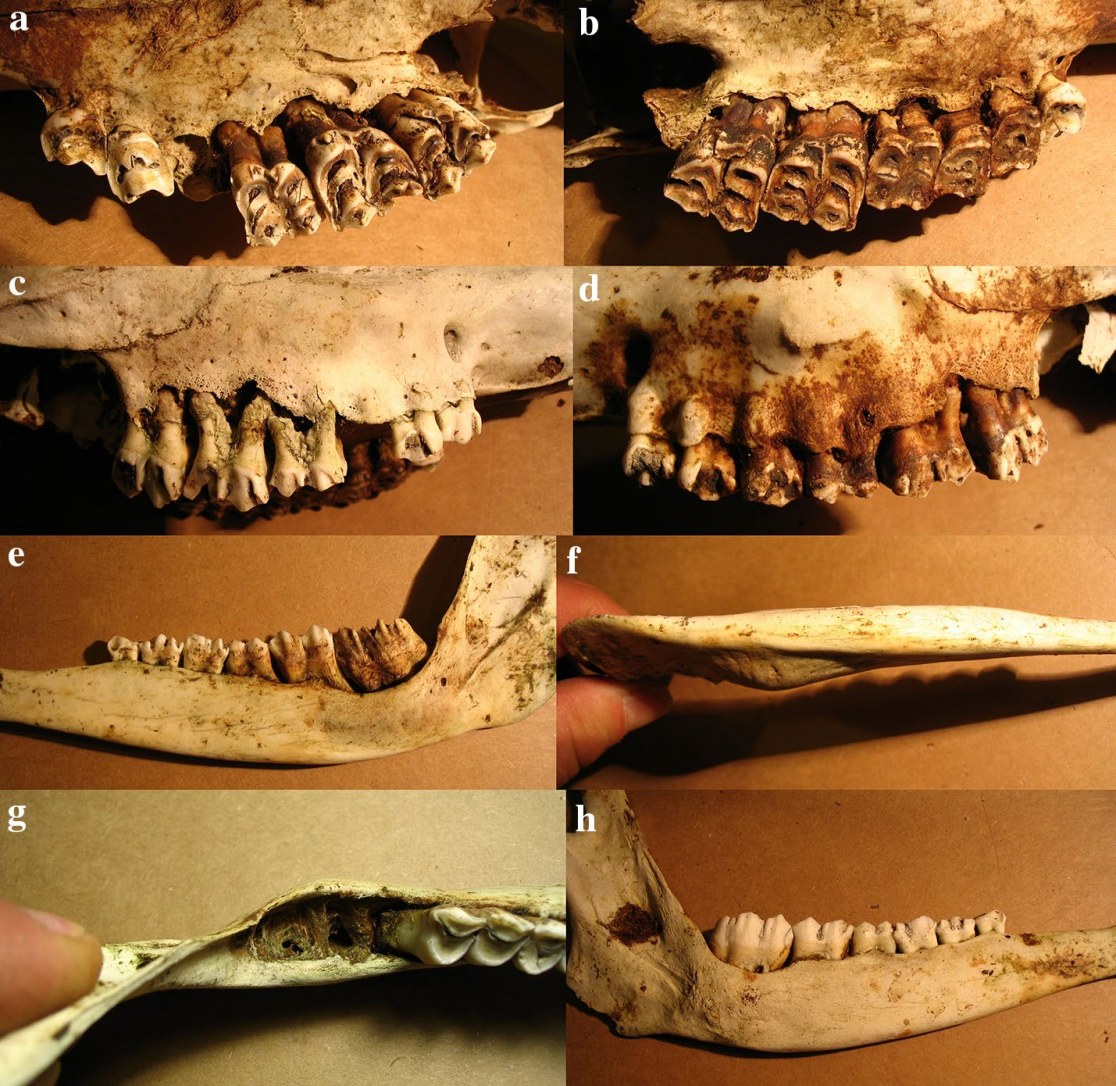
Case 3, a female 5–7 years old. **a** Erosions of the palate along the molars and of alveolar pockets. **b** Erosions and perforations of the palate. **c** Severe erosion of the right maxilla exposing molar roots, and perforations exposing premolar roots, similar to the left buccal side (**d**). **e** Advanced erosion and perforations at the level of molars, and a reduced jaw height. **f**, **g** Eroded alveolar pockets, missing molars, and mandibular thickening. **h** Erosions and reduced of mandibular height.

Case 3 is a skull from a female estimated at 5–7 years old based on tooth wear. The palatal side of the right maxilla shows erosion of alveolar pockets of P4 and erosion of the palate along the molars (Fig. 4a), whereas the left side has erosions and perforations of the palate (Fig. 4b). The right buccal side shows severe erosion of the maxilla exposing most of the roots of the molars, and with perforations exposing the roots of the premolars (Fig. 4c), similar to the left buccal side (Fig. 4d). The left buccal side of the mandible shows advanced erosion and perforations at the level of molars, and a reduced jaw height (Fig. 4e). The alveolar pockets at M3 are eroded while the jaw is thickened (Fig. 4f, g). The right buccal side of the mandible also exhibits erosion at M3 and a reduction of mandibular height (Fig. 4h).

**Fig. 5:**
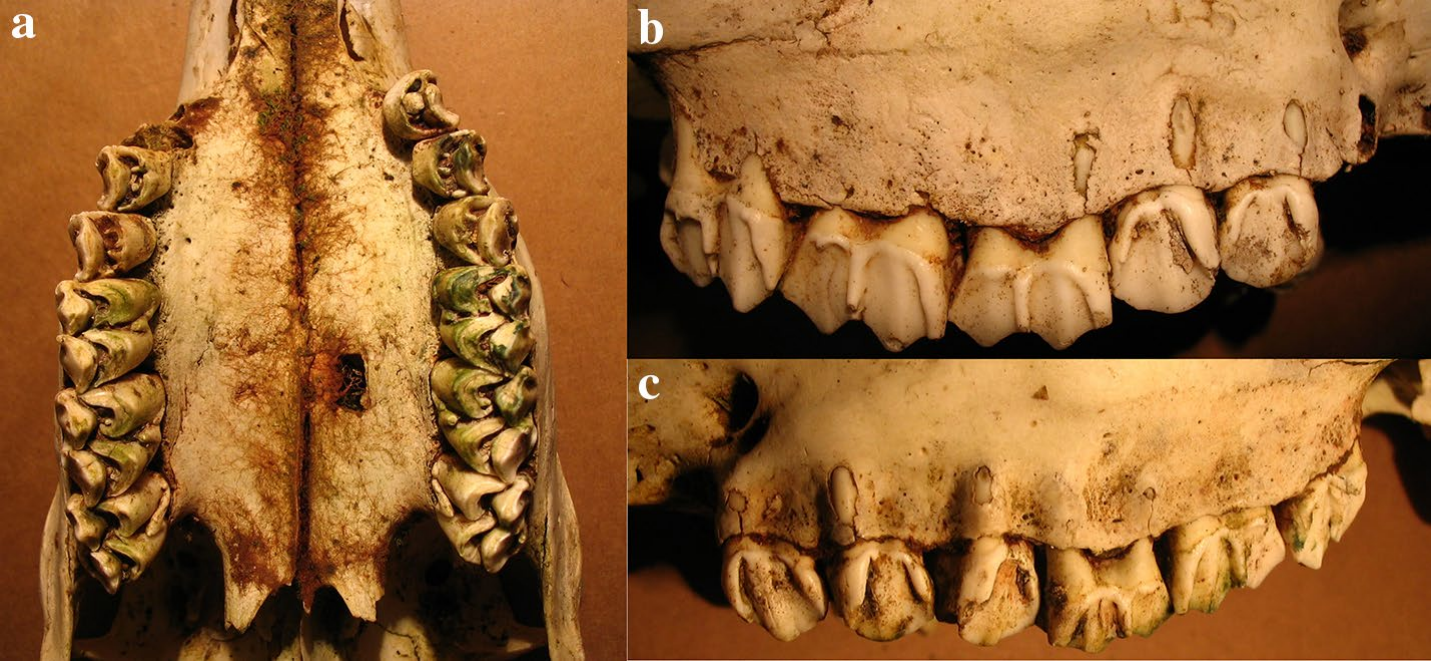
Case 4, a female 2–4 years old. **a** Porosification of palate and eroded alveolar pockets. **b** Maxillary buccal sides with porosification and perforations exposing root apices of most teeth.

Case 4 is a skull from a female estimated at 2–4 years old based on tooth wear, i.e. a young animal. The palate bordering the right premolars shows porosification and the alveoli of P4 are eroded (Fig. 5a). The left and right buccal sides of the maxilla show porosification and perforations which expose the roots of most upper teeth (Fig. 5b, c).

## Discussion

### Osteopathology in huemul both in Argentina and in Chile

The descriptions of pathological lesions encountered in cranial bones of huemul from the RNLC region coincide with those observed in specimens from Argentina [2]. The degree of the lesions from RNLC described here is considered severe and appears to surpass the cases from Argentina, like the present case 3 regarding maxillary lesions, or case 2 regarding mandibular lesions including a fractured main body. The prevalence in an Argentine population was at least 57%, whereas the data from the RNLC does not allow its estimation. Nonetheless, the encounter of the four described cases during a short study indicates that such osteopathology is not a rare event.

Regarding the potential etiological factors postulated by Flueck and Smith-Flueck in 2008 [2], fluorosis could be discarded for huemul in Argentina [10], which likely applies to the RNLC region as no reports of fluorosis in livestock nor people for that region were found. In contrast, the occurrence of iodine deficiency in southern Chile has been documented in people [28] and livestock [36, 37]. Primary iodine deficiency thus may also play a role in huemul, but in addition is aggravated by Se deficiency due to its key role in iodine metabolism. The occurrence of Se deficiency in huemul from RNLC has now been confirmed [22, 23] and coincides with other reports about Se deficient plants and livestock in southern Chile (reviewed in [43]). Soil samples from Argentina in areas used by extant huemul corroborate the deficient levels in forage, livestock and huemul by also revealing highly deficient levels [35].

### Are huemul from RNLC (Chile) deficient in selenium?

The alternative interpretation [23] of the first-ever study on Se status in huemul from RNLC related to the conclusion by Chihuailaf et al. [22] that “Se values were within the interval reported for white-tailed deer”, and that Se concentrations from huemul were “within reference intervals calculated for other deer”, referring to the study by [24]. However, the review by [24] was not based on data from deer “in one area” as asserted by Chihuailaf and Corti [44], but provided results from 8 different populations with means differing by up to 542%. These results certainly cannot serve as reference intervals, but mainly because [24] mentioned deer and livestock in their study areas as having been diagnosed with white muscle disease, and 50% of the deer were severely deficient and 75% had less than the critical concentration by livestock standards.

More importantly, we questioned the removal of 36% of data points from the analysis by [22], based on the claim that these were outliers. In response, Chihuailaf and Corti [44] only mention that Friedrichs et al. [45] suggest the removal of outliers to minimize error when establishing reference intervals. However, according to Friedrichs et al. definition of outliers we must ask: were these eliminated data points considered not to belong to the underlying distribution of the overall data set? or were they the result of preanalytical, analytical, and postanalytical errors? or did they result from erroneous inclusion of results from individuals that did not satisfy selection criteria? Instead, the samples eliminated from analysis by [22] were removed only because values were below the detection limit (bDL) of their equipment. Deleting these bDL data points resulted in a *type I* left-censored data set, an erroneous analysis and conclusions that Se levels were within reference intervals of other deer. However, these bDL data cannot be ignored nor deleted from subsequent analyses. Rather, commonly used substitution methods are replacement of bDL data by zero, or by half of DL, or the DL itself [46, 47], with DL/2 performing well in small samples [48]. By using the *conservative value* of DL for these previously discarded data, the huemul samples indicated 64% severely deficient, 9% deficient, and 18% marginal values [23].

Chihuailaf and Corti [44] further suggested that “North America, where acid rain produces soils low in selenium”, would be problematic for their wildlife, as opposed to southern South America which “lacks chronic atmospheric pollution”. This assertion does not hold up, first because only limited parts of North America with low soil Se are impacted by acid rain. Then to contradict this suggestion, [22] stated: “huemul currently inhabit mountainous ecosystems… those environments are formed on volcanic soils with pH tending to acidity”. In fact, data on soil pH of South America indicate that most all soils of the current huemul distribution are acidic (Fig. 1). Moreover, the fact that soils in areas with huemul are deficient in Se was highlighted by [23], averaging only 0.19 mg/kg of total Se [35]. These low soil Se levels and general acidic soil conditions best explain the well documented occurrence of Se deficiency in plants, livestock and huemul in southern Chile.

### The nexus between osteopathology in huemul and nutritional factors

The comprehensive literature on Se and iodine deficiency and their effects on physiology, including osteopathology, among many mammalian species, allows predictions on their effects on huemul. Moreover, basic mammalian biochemical functions demand a clear lower limit for Se provision to be able to provide adequate physiological responses. Due to remarkable interspecies similarities of the action of Se, there are few exceptions: direct extrapolations of Se requirements between species have a high degree of correlation [49]. This commonality has its roots in the fact that Se functions at very basic biochemical levels. First, the genetic code contains a codon which results in a Se-containing amino acid. Second, the rather recent discovery of the function of this codon revealed this new Se amino acid which was recognized as the 21st naturally occurring amino acid (reviewed in [25]). Thus, although the Se enzyme GSH-Px was hypothesized to exhibit higher activity per unit of blood Se in wild bighorn sheep (*Ovis canadensis*) than in domestic cattle, this however, was not the case [50]. In general, compared to non-ruminants, the dietary Se requirement is higher in ruminants, with more severity in small ruminants, due to lower diet Se absorption (ruminants: 11–35%, nonruminants: 77–85%: [51]). Specific circumstances may result in higher dietary requirements, such as the increased exposure to interfering elements like Cd, Hg or increased oxidative stress (reviewed in [25]). Moreover, subclinical effects may be the more frequent situation, yet these are best demonstrated mainly with production trials [52].

The argument that huemul have survived since the Pleistocene in habitats dominated by volcanic soils lacking in Se [44] needs qualification. First, during glaciation events, including the last glacial maximum, the area of the remaining extant huemul groups was under an ice cap reaching 1,800 m in thickness. Therefore, during these events, huemul in Patagonia existed only in areas east of the continental divide [3] and hence, far from soils dominated by volcanic ash. Moreover, animal species commonly occupy the landscape with strong presence in good habitat (source areas), but also areas that are marginal, and even areas incapable of sustaining populations (sink areas). The fact that most of the remaining >100 huemul groups fail to recover, or are diminishing, or have recently disappeared indicates that areas used by these extant huemul are not good enough as source areas. Lastly, the argument omits the crucial fact that the area with extant huemul contains a highly heterogeneous landscape due to the topography, yet huemul now are only using a very restricted portion which tends to be marginal or deficient regarding micronutrients. However, huemul formerly also utilized more fertile portions of this landscape particularly during winter, namely the places which became occupied with humans, their agriculture and livestock, or by towns and cities.

Several mineral nutrients like Se are not distributed homogeneously in the landscape, being consistently more concentrated in lower elevations in valley bottoms, flood plains, and in drier sites [30, 53]. Frequently, geological processes result in concentrations which become mineral licks used by wild ruminants, and these occur most often, even exclusively, in low elevations and on winter ranges [54, 55]. For instance, bighorn sheep made bimonthly short trips during the summer, to visit mineral licks at up to 2,000 m lower in elevation on traditional winter ranges, which replenished an otherwise Se-deficient summer diet [56]. Similarly, domestic ruminants have been shown to be Se deficient at high, but not at low elevation in the Columbian Andes, with Se enzyme activity differing by 41% [57]. Thus, if southern Chile has documented deficiencies of iodine and Se in livestock and humans living in the supposedly more fertile portion of the landscape, it follows that huemul in their restricted areas might be more compromised, as indicated by low survival of offspring [22], low longevity, and high prevalence of osteopathology [2]. Many of the >100 populations are thus declining, very few are increasing, and the species is numbering only about 1,500 individuals partitioned in over 100 groups.

## Conclusions

The lack of recovery of huemul must not be an enigma. The clinical severity of osteopathology in adults documented in Argentina (with the high prevalence of at least 57%), is now substantiated for huemul in the RNLC, Chile. Importantly, one of the hypothesized etiological factors playing a role has recently been confirmed in RNLC: Se deficiency. Moreover, other studies in southern Chile have already documented the occurrence of Se deficiency in livestock and deficient levels in forage plants. Lastly, Se levels in several soil samples from areas typically used by huemul in Argentina were recently shown to be at the *low end* of the range considered deficient. As the areas used currently by remaining huemul are documented to result in primary iodine deficiency, the Se deficiency additionally causes secondary iodine deficiency, such that the combined known effects on bone metabolism currently present the most parsimonious explanation for the osteopathology and lack of recovery. The nexus to the nutritional ecology of huemul likely is the inaccessibility of most lowlands and traditional winter ranges, the elimination of migratory traditions, and concomitant elimination of source populations.

In Argentina, an adaptive management approach is being undertaken by first determining the detailed spacial behavior of remnant populations of huemul to allow the strategic placement of salt blocks fortified with trace minerals like Se. Although there is important variability due to individual preference for using salt blocks, this approach has increased recruitment rates in north American wild herbivores [58, 59]. As many extant populations of huemul contain few individuals (10–20 individuals), another strategy would be to capture most individuals to provide them with commercial trace mineral rumen boluses which release minerals over about 3 years. This methods was used successfully in the 1980s on wild deer resulting in the recruitment rate increasing 2.6 fold [60].

## References

[1] Flueck WT, Smith-Flueck JM (2011). Osteological comparisons of appendicular skeletons: a case study on Patagonian huemul deer and its implications for conservation. Anim Prod Sci.

[2] Flueck WT, Smith-Flueck JM (2008). Age-independent osteopathology in skeletons of a south American cervid, the Patagonian huemul (*Hippocamelus bisulcus*). J Wildl Dis.

[3] Flueck WT, Smith-Flueck JM (2012). Huemul heresies: beliefs in search of supporting data. 1. Historical and zooarcheological considerations. Anim Prod Sci.

[4] Prichard HH (1902). Through the heart of Patagonia.

[5] Hatcher JB (1903). Reports of the Princeton University expeditions to Patagonia, 1896-1899. Vol. I: Narrative of the Expeditions. Geography of Southern Patagonia.

[6] Diaz NI (1993). Changes in the range distribution of *Hippocamelus bisulcus* in Patagonia. Z Säugetierkunde.

[7] Flueck WT, Smith-Flueck JM (2006). Predicaments of endangered huemul deer, *Hippocamelus bisulcus*, in Argentina: a review. Eur J Wildl Res.

[8] Krook LP, Justus C (2006). Fluoride poisoning of horses from artificially fluoridated drinking water. Fluoride.

[9] Araya O, Wittwer F, Villa A, Ducon C (1990). Bovine fluorosis following volcanic activity in the Southern Andes. Vet Rec.

[10] Flueck WT (2014). Continuing impacts on red deer from a volcanic eruption in 2011. Eur J Wildl Res.

[11] Garrott RA, Eberhardt LL, Otton JK, White PJ, Chaffee MA (2002). A geochemical trophic cascade in Yellowstone’s geothermal environments. Ecosystems.

[12] Schultz M, Kierdorf U, Sedlacek F, Kierdorf H (1998). Pathological bone changes in the mandibles of wild red deer (*Cervus elaphus* L.) exposed to high environmental levels of fluoride. J Anat.

[13] Moreno-Reyes R, Egrise D, Neve J, Pasteels JL, Schoutens A (2001). Selenium deficiency-induced growth retardation is associated with an impaired bone metabolism and osteopenia. J Bone Min Res.

[14] Köhrle J, Contempre B, Dumont JE, Jakob F (2005). Selenium, the thyroid, and the endocrine system. Endocrine Rev.

[15] Andrews ED, Hartley WJ, Grant AB (1968). Selenium-responsive diseases of animals in New Zealand. NZ Vet J.

[16] Porter WL, Scott RS, Manktelow BW (1970). The occurrence of paradontal disease in sheep in relation to superphosphate topdressing, stocking rate and other related factors. NZ Vet J.

[17] Ceballos A, Wittwer FG, Contreras PA, Quiroz E, Bohmwald HL (1999). Blood activity of glutathione peroxidase and its correlation with blood selenium concentration in grazing dairy cattle. Pesqui Agropecu Bras.

[18] Wittwer F, Araneda P, Ceballos A, Contreras PA, Andaur M, Bohmwald H (2002). Glutathion peroxidase activity (GSH-Px) in grazing dairy cattle in the south of Chile (IXth Region) and their relation with selenium contents in the forage. Arch Med Vet.

[19] Leyan V, Wittwer F, Contreras PA, Phil M, Kruze J (2004). Serum and colostrum immunoglobulin concentrations from selenium deficient cows and in the blood of their calves. Arch Med Vet.

[20] Contreras PA, Wittwer F, Matamoros R, Mayorga IM, van Schaik G (2004). Effect of grazing pasture with a low selenium content on the concentrations of triiodothyronine and thyroxine in serum, and GSH-Px activity in erythrocytes in cows in Chile. NZ Vet J.

[21] Contreras PA, Paredes E, Wittwer F, Carrillo S (2005). Clinical case: outbreak of white muscle disease or nutritional muscular dystrophy in calves. Rev Cient FCV-LUZ.

[22] Chihuailaf RH, Stevenson VB, Saucedo C, Corti P (2014). Blood mineral concentrations in the endangered huemul deer (*Hippocamelus bisulcus*) from Chilean Patagonia. J Wildl Dis.

[23] Flueck WT, Smith-Flueck JM, Mincher BJ, Winkel LHE (2014). An alternative interpretation of plasma selenium data from endangered Patagonian huemul deer (*Hippocamelus bisulcus*). J Wildl Dis.

[24] McDowell LR, Forrester DJ, Stephen BL, Scott DW, Wilkinson NS (1995). Selenium status of white-tailed deer in southern Florida. J Wildl Dis.

[25] Flueck WT, Smith-Flueck JM, Mionczynski J, Mincher BJ (2012). The implications of selenium deficiency for wild herbivore conservation, a review. Eur J Wildl Res.

[26] Geering HR, Cary EE, Jones LHP, Allaway WH (1968). Solubility and redox criteria for the possible forms of selenium in soils. Soil Sci Soc Amer Proc.

[27] Mikkelsen RL, Page AL, Bingham FT (1989). Factors affecting selenium accumulation by agricultural crops. Soil Sci Soc Amer Spec Publ.

[28] Kelly FC, Snedden WW (1960) Prevalence and geographical distribution of endemic goitre. In: Endemic goitre. Monograph Series 44. World Health Organization, Geneva, p 27–233 13752376

[29] Dunn JT, van der Haar F (1990) A practical guide to the correction of iodine deficiency. Technical Manual Nr. 3. International Council for Control of Iodine Deficiency Disorders, UNICEF, WHO, Geneva

[30] Carter DL, Robbins CW, Brown MJ (1970). Selenium concentrations in forage on some high northwestern ranges. J Range Manage.

[31] Gupta UC, Gupta SC, Gupta MD (2000). Selenium in soils and crops, its deficiencies in livestock and humans: implications for management. Comm Soil Sci Plant Anal.

[32] Fordyce FM, Selinus O (2005). Selenium deficiency and toxicity in the environment. Essentials of medical geology: impacts of the natural environment on public health.

[33] Floor GH, Roman-Ross G (2012). Selenium in volcanic environments: a review. Appl Geochem.

[34] Lenz M, Floor GH, Winkel LH, Roman-Ross G, Corvini PF (2012). Online reconcentration-IC-ICP-MS for selenium quantification and speciation at ultratraces. Environ Sci Technol.

[35] Flueck WT, Smith-Flueck JM, Mincher BJ, Winkel LHE, Ma J, Zhang M, Halbrook R, Liu B, Zhang W (2014). Soil selenium levels corroborate direct evidence of selenium deficiency in endangered Patagonian huemul deer (*Hippocamelus bisulcus*). Proceedings of the 8th International Deer Biology Congress.

[36] Contreras PA, Ceballos A, Matamoros R, Wittwer F (2003). Iodine concentration in forages from dairy farms in the IXth and X-th Regions of Chile. Arch Med Vet.

[37] Matamoros R, Contreras PA, Wittwer F, Mayorga MI (2003). Hypothyroidism in ruminants. Arch Med Vet.

[38] Salvaneschi JP, García JR (2009). El bocio endémico en la República Argentina. Antecedentes, extensión y magnitud de la endemia, antes y después del empleo de la sal enriquecida con yodo. Primera parte. Rev Argent Endocrinol Metab.

[39] Arthur JR, Beckett GJ, Mitchell JH (1999). The interactions between selenium and iodine deficiencies in man and animals. Nutr Res Rev.

[40] Beckett GJ, Arthur JR (2005). Selenium and endocrine systems. J Endocrinol.

[41] Voudouri AE, Chadio SE, Menegatos JG, Zervas GP, Nicol F, Arthur JR (2003). Selenoenzyme activities in selenium- and iodine-deficient sheep. Biol Trace Elem Res.

[42] Pavlata L, Prasek J, Filipek J, Pechova A (2004). Influence of parenteral administration of selenium and vitamin E during pregnancy on selected metabolic parameters and colostrum quality in dairy cows at parturition. Vet Med Czech.

[43] Flueck WT, Smith-Flueck JM (2011). Recent advances in the nutritional ecology of the Patagonian huemul: implications for recovery. Anim Prod Sci.

[44] Chihuailaf RH, Corti P (2014). Interpretation of plasma selenium data in huemul: response to Flueck et al. J Wildl Dis.

[45] Friedrichs KR, Harr KE, Freeman KP, Szladovits B, Walton RM, Barnhart KF (2012). ASVCP reference interval guidelines: determination of de novo reference intervals in veterinary species and other related topics. Vet Clin Pathol.

[46] Singh A, Nocerino J (2002). Robust estimation of mean and variance using environmental data sets with below detection limit observations. Chemometr Intell Lab Syst.

[47] Palarea-Albaladejoa J, Martin-Fernandez JA (2013). Values below detection limit in compositional chemical data. Anal Chim Acta.

[48] Clarke JU (1998). Evaluation of censored data methods to allow statistical comparisons among very small samples with below detection limit observations. Environ Sci Technol.

[49] Koller LD, Exon JH (1986). The two faces of selenium—deficiency and toxicity—are similar in animals and man. Can J Vet Res.

[50] Samson J, Jorgenson JT, Wishart WD (1989). Glutathione peroxidase activity and selenium levels in Rocky Mountain bighorn sheep and mountain goats. Can J Zool.

[51] Hefnawy AEG, Tórtora-Pérez JL (2010). The importance of selenium and the effects of its deficiency in animal health. Small Ruminant Res.

[52] Suttle N (2010). Mineral nutrition of livestock.

[53] Ren JZ, Zhou ZY, Pan B, Chen W, Comb GF, Spallholz JE, Levander OA, Oldfield JE (1987). Selenium distribution in four grassland classes of China. Selenium in biology and medicine.

[54] McCann LJ (1956). Ecology of the mountain sheep. Am Midl Nat.

[55] Poole KG, Bachmann KD, Teske IE (2010). Mineral lick use by GPS radio-collared Mountain goats in Southeastern British Columbia. West North Am Nat.

[56] Mincher BJ, Mionczynski J, Hnilicka PA, Ball RD, Houghton TX (2008). Some aspects of geophagia in Wyoming bighorn sheep (*Ovis canadensis*). Eur J Wildl Res.

[57] Jaramillo S, Villa NA, Pineda AF, Gallego AB, Tabares P, Ceballos A (2005). Actividad sanguínea de superóxido dismutasa y glutatión peroxidasa en novillas a pastoreo. Pesqui Agropecu Bras.

[58] Hnilicka PA, Mionczynski J, Mincher BJ, States J, Hinschberger M, Oberlie S (2004). Bighorn sheep lamb survival, trace minerals, rainfall, and air pollution: are there any connections?. Bienn Symp North Wild Sheep Goat Council.

[59] Coggins VL (2006). Selenium supplementation, parasite treatment, and management of Bighorn sheep at Lostine River, Oregon. Bienn Symp North Wild Sheep Goat Council.

[60] Flueck WT (1994). Effect of trace elements on population dynamics: selenium deficiency in free-ranging black-tailed deer. Ecology.

